# The prediction of early progressive disease in patients with hepatocellular carcinoma receiving atezolizumab plus bevacizumab

**DOI:** 10.1002/cam4.6369

**Published:** 2023-08-03

**Authors:** Yasuto Takeuchi, Kazuhiro Nouso, Shin‐ichi Fujioka, Kazuya Kariyama, Haruhiko Kobashi, Shuji Uematsu, Akio Moriya, Hiroaki Hagihara, Hiroyuki Takabatake, Shinichiro Nakamura, Kazuhisa Yabushita, Tatsuya Kikuchi, Atsushi Oyama, Takuya Adachi, Nozomu Wada, Hideki Onishi, Hidenori Shiraha, Akinobu Takaki

**Affiliations:** ^1^ Department of Regenerative Medicine, Center for Innovative Clinical Medicine Okayama University Hospital Okayama Japan; ^2^ Department of Gastroenterology and Hepatology Okayama University Graduate School of Medicine, Dentistry and Pharmaceutical Sciences Okayama Japan; ^3^ Department of Gastroenterology Okayama City Hospital Okayama Japan; ^4^ Department of Gastroenterology Okayama Saiseikai General Hospital Okayama Japan; ^5^ Department of Gastroenterology Japanese Red Cross Okayama Hospital Okayama Japan; ^6^ Department of Gastroenterology Hiroshima City Hospital Okayama Japan; ^7^ Department of Gastroenterology Mitoyo General Hospital Okayama Japan; ^8^ Department of Gastroenterology Sumitomo Besshi Hospital Okayama Japan; ^9^ Department of Gastroenterology Kurashiki Central Hospital Okayama Japan; ^10^ Department of Gastroenterology Japanese Red Cross Himeji Hospital Okayama Japan; ^11^ Department of Gastroenterology Fukuyama City Hospital Okayama Japan

**Keywords:** atezolizumab, bevacizumab, early progression, hepatocellular carcinoma, real‐world practice

## Abstract

**Background and Aims:**

The IMbrave 150 trial revealed the usefulness of atezolizumab plus bevacizumab therapy in patients with unresectable hepatocellular carcinoma (HCC), making it now considered the first‐line systemic chemotherapy agent for HCC. The present study investigated factors associated with early tumor progression of atezolizumab plus bevacizumab in patients with advanced HCC in real‐world clinical practice.

**Methods:**

A total of 184 HCC patients who received atezolizumab plus bevacizumab therapy were studied. We investigated the frequency of early progressive disease (e‐PD; PD within 9 weeks) and analyzed the risk factors for e‐PD.

**Results:**

There were 47 patients (25.5%) diagnosed as e‐PD. Patients with e‐PD had a worse performance status (PS) and albumin–bilirubin (ALBI) and Child‐Pugh (C‐P) scores and a significantly higher rate of a systemic therapy than those with non‐e‐PD. A multivariate analysis showed that PS ≥1 (odds ratio [OR] = 4.5, 95% confidence interval [CI] = 1.9–10, *p* < 0.001), ALBI score ≥−2.30 (OR = 2.1, 95% CI = 1.0–4.5, *p* = 0.044) and the history of a systemic therapy (OR = 3.0, 95% CI = 1.4–6.4, *p* = 0.0038) were significant and independent determinants of e‐PD. When examining the liver function trends in e‐PD patients, the ALBI scores at 3 and 6 weeks after starting therapy were significantly higher than before the treatment (*p* < 0.001).

**Conclusions:**

The liver function and systemic therapy are useful predictors of e‐PD in HCC patients treated with atezolizumab plus bevacizumab in real‐world clinical practice.

## 1 INTRODUCTION

Hepatocellular carcinoma (HCC) continues to be one of the most prevalent cancers,^1^ making it a major health problem around the world. For a long time, local therapies, such as radiofrequency ablation and transcatheter arterial chemoembolization, have been the main treatment modalities for unresectable HCC, but there are many cases where treatment becomes difficult due to disease progression. Systemic chemotherapy has been aggressively introduced for such cases. In Japan, sorafenib was approved as the molecular‐targeted agent in 2009, followed by regorafenib, ramucirumab, lenvatinib, and cabozantinib. The sixth regimen, atezolizumab plus bevacizumab, was approved in 2020 and is now widely accepted as the first‐line systemic chemotherapy for advanced HCC. The advent of these agents has improved the prognosis of patients with advanced HCC.^2^

Atezolizumab selectively targets PD‐L1 and prevent PD‐1 and B7‐1 receptor interactions associated with T‐cell suppression.^3^ Bevacizumab is a monoclonal antibody that inhibits the action of vascular endothelial growth factor (VEGF), thus suppressing angiogenesis and tumor growth and metastasis.^4^, ^5^ HCC is hypervascular tumor, and such angiogenesis is activated by VEGF. In addition, VEGF is also related in cancer immune evasion mechanisms. Combination therapy of targeting PD‐L1 (atezolizumab) and VEGF (bevacizumab) is thus considered the effective therapy for advanced HCC.

However, as experience with their use in real clinical practice has increased, it has become clear that a certain number of patients do not respond to treatment and develop progressive disease at an early stage (e‐PD). Although activation of WNT/β‐catenin that occurs in 20% of HCC is considered the cause of the refractoriness,^6^ the characteristics of patients who do not respond to the treatment and develop e‐PD have not yet been fully clarified.

We therefore investigated the frequency of e‐PD and its characteristics in order to select appropriate treatment in patients with advanced HCC.

## 2 METHODS

### 2.1 Patients

Two hundred and five patients with HCC receiving atezolizumab plus bevacizumab therapy who were treated at Okayama University Hospital or its affiliated hospitals between 2020 and 2022 were registered. Among these 205 patients, 15 without imaging findings at 6–9 weeks after the start of treatment and 6 who were not assessable were excluded. Ultimately, 184 patients were analyzed.

The HCC diagnosis and evaluation of disease progression were based on the guidelines proposed by the Japan Society of Hepatology,^7^ American Association for the Study of Liver Disease,^8^ and European Association for the Study of the Liver.^9^

### 2.2 Atezolizumab plus bevacizumab therapy regimens

All patients received atezolizumab (1200 mg) plus bevacizumab (15 mg/kg) therapy every 3 weeks until unacceptable adverse events (AEs) or disease progression occurred. Treatment interruption or dosage reduction were performed based on the guidelines. Patients continued atezolizumab plus bevacizumab therapy until death or if one of the following criteria was met: occurrence of AEs above Grade 3, progressive disease following treatments, deterioration of Eastern Cooperative Oncology Group Performance Status to 4, a worsening liver function (Child‐Pugh [C‐P] score ≥8), or withdrawal of consent.

### 2.3 Follow‐up and the assessment of the response to therapy

The characteristics of the patients, including their hematological, biochemical, and virological data, were collected at enrollment. After atezolizumab plus bevacizumab administration, the patients were followed every 3 weeks continuously. Imaging studies for HCC, such as dynamic computed tomography or magnetic resonance imaging, were performed at baseline, 6–9 weeks after atezolizumab plus bevacizumab administration, and every 6–12 weeks thereafter. Treatment response was judged based on the Response Evaluation Criteria in Solid Tumors (RECIST)^10^ version 1.1. AEs were evaluated according to the Common Terminology Criteria for Adverse Events version 5.0. The alpha fetoprotein (AFP) and des‐gamma‐carboxy prothrombin (DCP) level were evaluated at baseline and every 3 weeks thereafter. To assess changes in the liver function, the C‐P score and albumin–bilirubin (ALBI) score^11^, ^12^ were analyzed at baseline, 3 and 6 weeks after treatment, and the point of disease progression.

### 2.4 Definition of e‐PD

Target lesions were selected from up to five large lesions that were measurable on imaging prior to treatment initiation, based on RECIST 1.1. e‐PD was defined as an increase of at least 20% in the diameter sum of the target lesion relative to the smallest diameter sum during the course (if the baseline diameter sum was the smallest during the course, it was considered the smallest diameter sum) and an absolute increase of at least 5 mm in the diameter sum, within 9 weeks after atezolizumab plus bevacizumab administration, according to RECIST 1.1.^10^

### 2.5 Statistical analyses

Data were expressed as the median (range). The statistical analyses included the Fisher's exact test or chi‐squared test and Mann–Whitney *U*‐test. The transition of the liver function was examined by a paired *t*‐test. A logistic regression analysis was used to predict the factors associated with treatment response after atezolizumab plus bevacizumab therapy. The cutoff value was defined as the median for age, ALBI score, AFP, and DCP. The progression‐free survival (PFS) and overall survival (OS) were estimated by the Kaplan–Meier method and compared among the patient groups using the log‐rank test. *p* values <0.05 were considered significant.

The statistical analyses were performed using the JMP software program, version 16 (SAS Institute, Cary, NC, USA).

## 3 RESULTS

### 3.1 Tumor responses and patient characteristics

**FIGURE 1:**
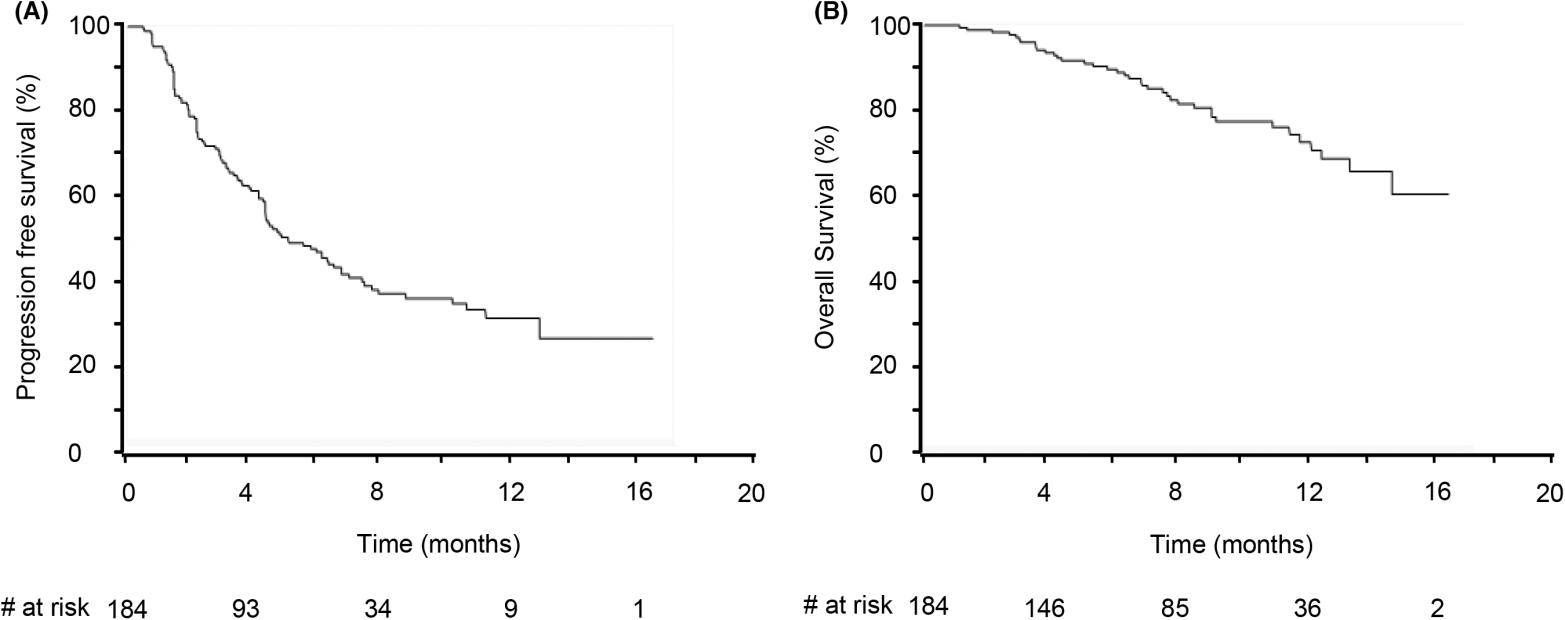
The progression‐free survival (A) and overall survival (B) in all patients. The median progression‐free survival was 4.9 months. The median overall survival was not reached during the observation period, and the 1‐year survival rate was 68.2%.

Based on the best treatment effect during the treatment period, the proportion of patients with a complete response (CR), partial response (PR), stable disease (SD), and progressive disease (PD) was 2.2%, 27.7%, 41.3%, and 28.8%, respectively. The objective response rate (ORR; CR + PR) was 29.9%, and the disease control rate (DCR; CR + PR + SD) was 71.2%. Both the ORR and DCR were significantly higher in the first‐line patients than in the later‐line patients (Table 1). The median PFS was 4.9 months, and the median OS was not reached during the observation period (Figure 1).

**TABLE 1:** Tumor responses based on RECIST 1.1.

Response	All patients (*n* = 184)	First‐line patients (*n* = 119)	Later line patients (*n* = 65)	*p* value
Objective response rate	55 (29.9%)	45 (37.8%)	10 (15.4%)	0.0020
Disease control rate	131 (71.2%)	99 (83.2%)	32 (49.2%)	<0.001
Complete response	4 (2.2%)	3 (2.5%)	1 (1.5%)	NS
Partial response	51 (27.7%)	42 (35.3%)	9 (13.8%)	<0.001
Stable disease	76 (41.3%)	54 (45.4%)	22 (33.8%)	<0.001
Progressive disease	53 (28.8%)	20 (16.8%)	33 (50.8%)	NS

Abbreviation: NS, not significant.

The characteristics of the patients at initiation of atezolizumab plus bevacizumab therapy are shown in Table 2. The median age was 74 years old, and 153 patients (84.1%) were male. A hundred patients (55.0%) had a C‐P score of 5, and the median ALBI score was −2.35. Atezolizumab plus bevacizumab were used as first‐line systemic therapy in 117 patients (64.3%), and 65 patients (35.7%) had a history of other systemic chemotherapy. The details of pretreatment in these 65 patients are shown in Table 3. This group included 48 patients treated as second‐line, 9 as third‐line, 7 as forth‐line and 1 as fifth‐line. In patients with systemic therapy, liver function was significantly impaired at the start of atezolizumab plus bevacizumab therapy (−2.20 and −2.39, respectively, *p* = 0.0041).

**TABLE 2:** Characteristics of the patients enrolled in the present study.

Patient characteristics	All patients (*n* = 182)	e‐PD (*n* = 50)	Non‐e‐PD (*n* = 132)	*p* value
Age (years)	74 (30–92)^+^	76 (53–88)^+^	73 (30–92)^+^	NS
Gender				
Male	153 (84.1%)	43 (86.0%)	110 (83.3%)	NS
Female	29 (16.0%)	7 (14.0%)	22 (16.7%)	
Etiology				
Viral	91 (50.0%)	24 (48.0%)	67 (50.8%)	NS
Nonviral	91 (50.0%)	26 (52.0%)	65 (49.2%)	
Child‐Pugh score				
5	100 (55.0%)	21 (42.0%)	79 (59.8%)	0.030
≥6	82 (45.0%)	29 (58.0%)	53 (40.2%)	
Performance status				
0	144 (79.1%)	29 (58.0%)	115 (87.1%)	<0.001
1	38 (20.8%)	21 (42.0%)	17 (12.9%)	
ALBI score	−2.35 (−3.32–1.34)^+^	−2.13 (−3.33–1.34)^+^	−2.40 (−3.17–1.58)^+^	<0.001
BCLC stage				
A	9 (5.0%)	1 (2.0%)	8 (6.1%)	NS
B	63 (34.8%)	12 (24.0%)	51 (38.9%)	
C	109 (60.2%)	37 (74.0%)	72 (55.0%)	
Macrovascular invasion				
Present	45 (24.9%)	9 (18.0%)	36 (27.5%)	NS
Absent	136 (75.1%)	41 (82.0%)	95 (72.5%)	
Extrahepatic metastasis				
Present	56 (30.9%)	21 (42.0%)	35 (26.7%)	NS
Absent	125 (69.1%)	29 (58.0%)	96 (73.3%)	
AFP (ng/mL)	43.7 (1.1–158,488)^+^	80 (1.7–20,000)^+^	32.6 (1.1–880,335)^+^	NS
AFP‐L3 (%)	23.1 (0–99.5)^+^	24.3 (0–99.5)^+^	20.5 (0–88.9)^+^	NS
DCP (mAU/mL)	431.9 (8.8–673,927)^+^	765 (18–673,927) ^+^	332 (8.8–243,219)^+^	NS
Past systemic chemotherapy				
Present	65 (35.7%)	27 (54.0%)	38 (28.8%)	<0.001
Absent	117 (64.3%)	23 (46.0%)	94 (71.2%)	

*Note*: Data are presented as the median (range) indicated + sign or *n* (%) unless otherwise indicated.

Abbreviations: AFP, alpha‐fetoprotein; AFP‐L3, LCA‐reactive alpha‐fetoprotein isoform; ALBI, albumin–bilirubin; CLC, Barcelona clinic liver cancer; DCP, des‐gamma‐carboxy prothrombin; NS, not significant.

**TABLE 3:** Treatment of patients with a systemic therapy.

Line	Treatment	*n*
Second line	Lenvatinib	42
	Sorafenib	6
Third line	Sorafenib/regorafenib	3
	Sorafenib/lenvatinib	4
	Lenvatinib/ramucirumab	2
Fourth line	Sorafenib/regorafenib/lenvatinib	4
	Sorafenib/lenvatinib/ramucirumab	2
	Lenvatinib/ramucirumab/cabozantinib	1
Fifth line	Lenvatinib/sorafenib/ramucirumab/regorafenib	1

**FIGURE 2:**
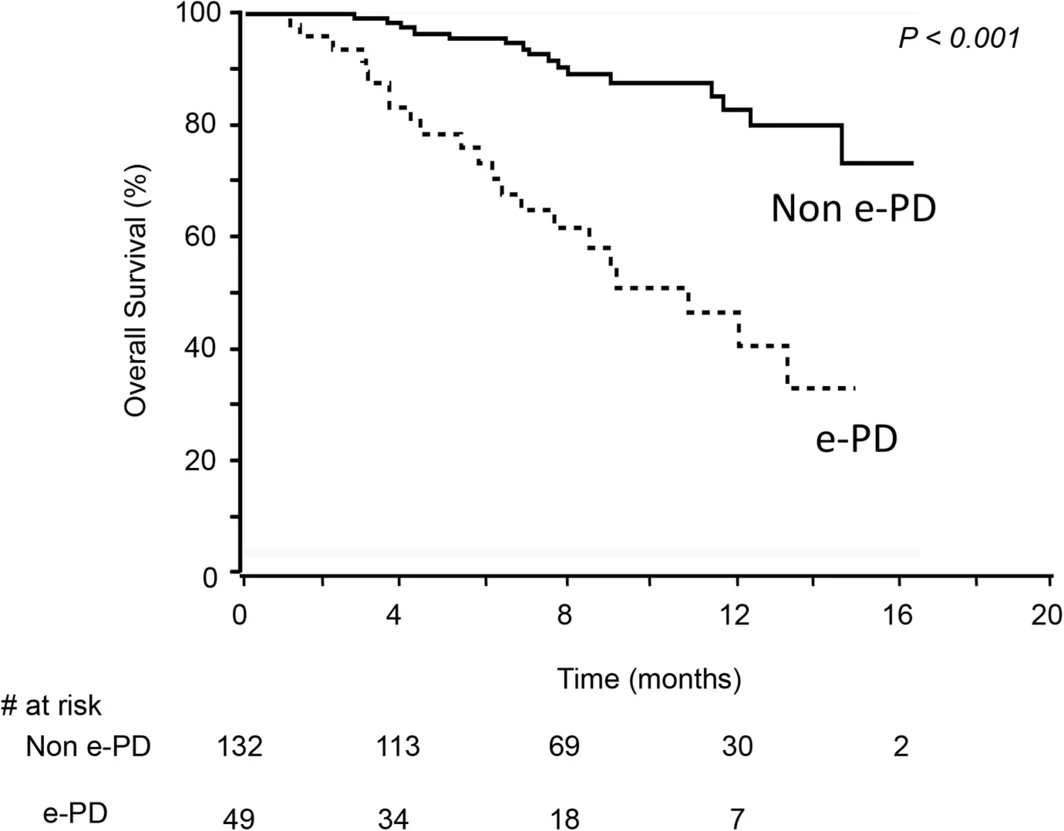
The comparison of the overall survival in e‐PD and non‐e‐PD patients. Patients with e‐PD showed a significantly lower overall survival rate than those without e‐PD (*p* < 0.001).

There were 50 patients (27.2%) diagnosed as e‐PD. The baseline PS, ALBI score and C‐P score were poorer in e‐PD patients than in non‐e‐PD patients, and the e‐PD patients showed a significantly higher rate of a systemic therapy than the non‐e‐PD patients (Table 1). In addition, as shown in Figure 2, there was a significant difference in the OS between the e‐PD and non‐e‐PD patients. The median survival time was not reached in either group during the observation period. Sequential therapies were performed in 61 (33.2%) patients. The rates in e‐PD and non‐e‐PD groups were 51.1% (24 patients) and 36.6% (37 patients), respectively, and the difference was not statistically significant (*p* = 0.10, Table S1).

### 3.2 Risk factors for e‐PD

Table 4 shows the analysis of predictive factors for e‐PD using logistic regression. A univariate analysis showed that PS ≥1, ALBI score ≥−2.30, BCLC‐C and a systemic therapy were significant factors associated with e‐PD. A multivariate analysis showed that PS ≥1 (odds ratio [OR] = 4.5, 95% confidence interval [CI] = 1.9–10, *p* < 0.001), ALBI score ≥−2.30 (OR = 2.1, 95% CI = 1.0–4.5; *p* = 0.044), and a systemic therapy (OR = 3.0, 95% CI = 1.4–6.4, *p* = 0.0038) were significant, independent determinants of e‐PD. Neither adverse events nor dose reduction or withdrawal of bevacizumab within the first 9 weeks of the treatment was associated with e‐PD.

**TABLE 4:** Results of a logistic regression analysis of the factors associated with e‐PD.

Factors	Univariate analysis		Multivariate analysis	
	Odds ratio (95% CI)	*p* value	Odds ratio (95% CI)	*p* value
Age (≥75 years)	1.6 (0.80–3.0)	0.18		
Etiology (nonviral)	0.9 (0.48–1.8)	0.83		
Performance status (≥1)	4.9 (2.3–10)	<0.001	4.5 (1.9–10)	<0.001
ALBI score (≥−2.30)	2.6 (1.3–5.1)	0.006	2.1 (1.0–4.5)	0.044
Tumor size (≥30 mm)	1.4 (0.68–2.8)	0.37		
Tumor number (>3)	1.3 (0.60–2.64)	0.54		
Macrovascular invasion (present)	1.6 (0.72–3.7)	0.23		
Main portal vein invasion (present)	1.7 (0.53–4.3)	0.23		
Extrahepatic metastasis (present)	1.6 (0.82–3.3)	0.16		
BCLC (C)	2.3 (1.1–4.8)	0.029	1.3 (0.56–3.0)	0.54
AFP (≥400 ng/mL)	1.3 (0.62–2.6)	0.52		
AFP‐L3 (≥25%)	1.1 (0.51–2.2)	0.89		
DCP (≥400 mAU/mL)	1.6 (0.83–3.2)	0.15		
AE within 9 weeks (present)	0.9 (0.39–1.9)	0.68		
Reduction or withdrawal of bevacizumab within 9 weeks (present)	1.1 (0.37–3.3)	0.85		
Systemic therapy (present)	3.1 (1.6–6.2)	0.001	3.0 (1.4–6.4)	0.003

Abbreviations: AE, adverse event; AFP, alpha‐fetoprotein; AFP‐L3, LCA‐reactive alpha‐fetoprotein isoform; ALBI, albumin–bilirubin; CI, confidence interval; DCP, des‐gamma‐carboxy prothrombin.

In patients who did not have a systemic therapy, a univariate analysis showed that PS ≥1, NAFLD etiology and BCLC‐C were significant factors associated with e‐PD. But multivariate analysis showed no independent determinants of e‐PD (Table S2).

### 3.3 Liver function trends after treatment

**FIGURE 3:**
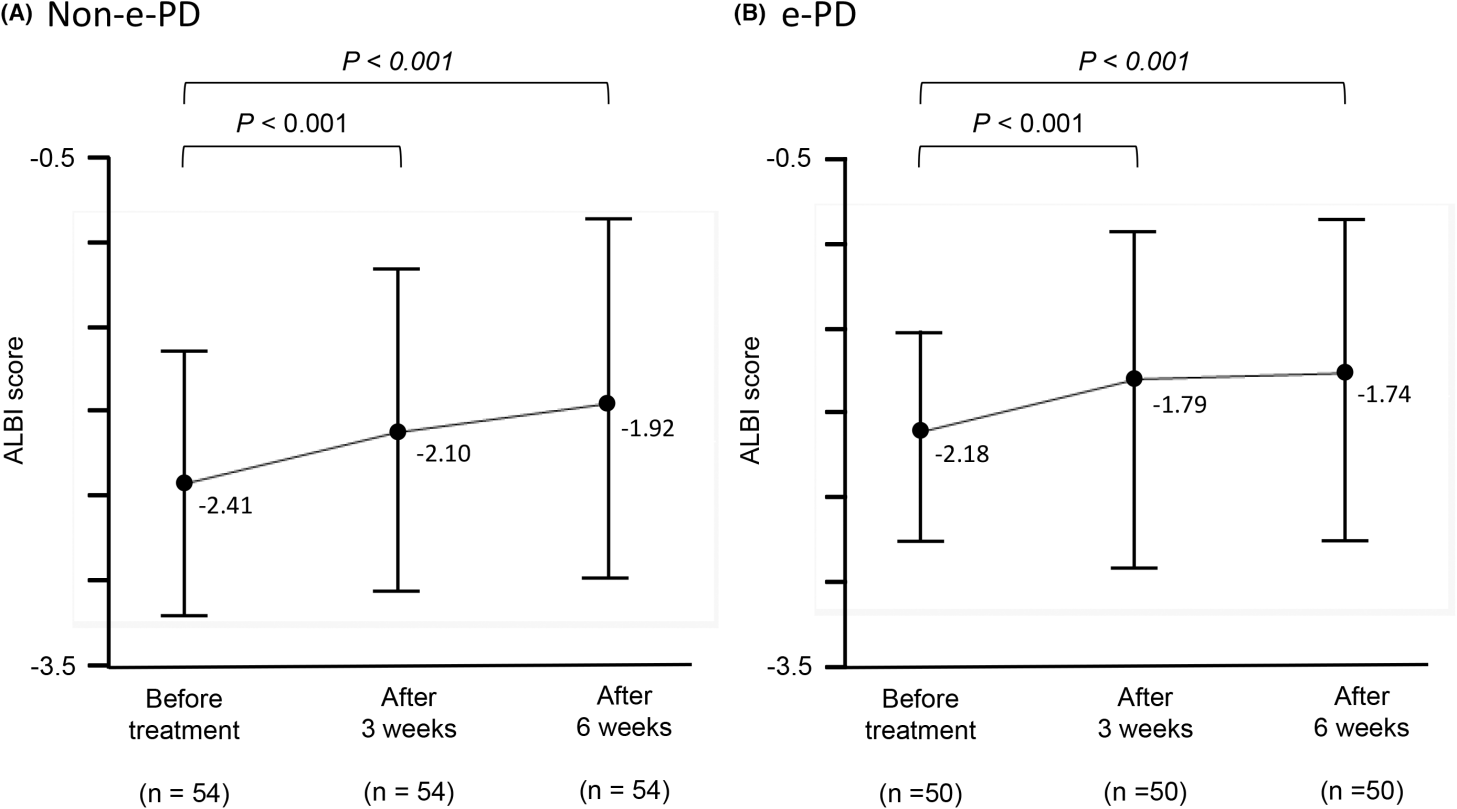
Changes in the ALBI score after treatment initiation. In non‐e‐PD patients, a significant increase in the ALBI score was observed at 3 weeks after the start of treatment (*p* < 0.001), remaining roughly the same at 6 weeks (A). Patients with e‐PD also showed a significant increase in the ALBI score in the first 3 weeks of treatment (*p* < 0.001), and their liver function remained deteriorated at 6 weeks (B).

The liver function after atezolizumab plus bevacizumab treatment is shown in Figure 3. In non‐e‐PD patients, ALBI scores had worsened significantly at 3 weeks compared with the baseline (*p* < 0.001) and remained similar at 6 weeks. A similar trend was also observed in e‐PD patients, with ALBI scores worsening significantly (*p* < 0.001) in the first 3 weeks of treatment and the liver function remaining poor at 6 weeks.

## 4 DISCUSSION

Systemic chemotherapy in the treatment of HCC has advanced remarkably, and at present, three regimens are available as first‐line therapy in Japan: sorafenib, lenvatinib, and atezolizumab plus bevacizumab. The high efficacy results of the previous IMbrave150 have allowed atezolizumab plus bevacizumab to be considered the first‐line treatment in treatment guidelines. The present study revealed that e‐PD is observed in approximately 25% of patients who receive atezolizumab plus bevacizumab. e‐PD patients showed a higher rate of a systemic therapy than those without e‐PD, and their PS and liver function were significantly worse than in those without e‐PD. These factors were also shown to be associated with e‐PD by a multivariate analysis.

There have been several reports examining factors related to the efficacy of atezolizumab plus bevacizumab in patients with HCC. β‐catenin (CTNNB1) gene mutations are reported to be frequently found in HCC. In the presence of β‐catenin mutations, there is no intratumoral infiltration of CD8‐positive T cells, which attack cancer cells, so immune checkpoint inhibitors (ICIs) may not be effective.^13^ And PD‐1/PD‐L1 protein expression and tumor mutation burden‐high (TMB‐H) have been reported as biomarkers of the efficacy of anti‐PD‐1/PD‐L1 antibody drugs.^14^, ^15^ However, while both markers may be useful in predicting the treatment response, their utility for predicting e‐PD has not been studied. In addition, it is difficult to measure these markers routinely in real clinical practice because they require a liver biopsy. Nonalcoholic steatohepatitis (NASH) might be one reason for the resistance to immunotherapeutic drugs.^16^ Immune responses to tumor antigens were reported to be lower in patients with NASH than in those with viral HCC,^17^ and the presence of CD8‐positive regulatory T cells, which are frequently expressed in NASH, was considered the main reason for this phenomenon.^18^ However, this trend was not confirmed in the present study. Notably, there have been several reports stating that ICIs are more likely to be effective in NASH‐HCC when combined with a molecular‐targeted agent, such as bevacizumab,^19^ which was consistent with the results in this study. The usefulness of the chronological measurement of AFP as a predictor of response to atezolizumab plus bevacizumab therapy has also been reported.^20^, ^21^ However, while the measurement method is noninvasive, the ability of AFP to predict e‐PD has not been studied in detail.

In the present study, we focused on e‐PD for the first time. The noninvasive prediction of e‐PD before treatment is important for making early decisions to change treatment regimens and hopefully extend the patient's survival.

Our findings suggested an association between a systemic therapy and e‐PD. The results were similar to the previous report by Hiraoka et al. In the report, PD rates in patients with a systemic therapy was higher than that in patients without the therapy (22.2% and 17.1%, respectively),^22^ although the difference was not statistically significant (*p* = 0.156). Indeed, this relationship was not studied even in the IMbrave150 trial, as patients with a history of therapy were excluded.^23^ Why systemic therapies correlated with e‐PD is unclear at present; however, there are several possible explanations. Although there have been no reports that clearly explained the mechanism of the association between a history of therapy and treatment response, it is possible that long‐term use of sorafenib or lenvatinib may have led to resistance to molecular‐targeted agents, thereby reducing the efficacy of bevacizumab and resulting in e‐PD.

In this study, liver function before treatment was an independent predictor of e‐PD. Previous report showed the similar results that the efficacy of lenvatinib in patients with good liver function was better than in patients with poor liver function.^24^ The result is similar to the past reports which showed the prognostic impact of liver function in HCC. Those reports have been well documented in not only retrospective studies, but also in analyses of the phase‐III trials of sorafenib, cabozantinib, ramucirumab, or regorafenib in advanced HCC.^25^, ^26^, ^27^, ^28^, ^29^

In the present study, worsening of the ALBI score with atezolizumab plus bevacizumab was observed in both non‐e‐PD and e‐PD patients. In both groups, the ALBI score increased significantly during the first 3 weeks of treatment and did not change thereafter. Liver dysfunction due to the use of molecular‐targeted drugs has been reported.^30^, ^31^ Although this finding was not demonstrated in the IMbrave150 trial, temporal liver dysfunction at an early stage of the combination therapy regardless of e‐PD or non‐e‐PD was shown in another study.^22^ The reason of the liver dysfunction in both non‐e‐PD and e‐PD patients has not been elucidated well; however, decrease of hepatic blood flow or appetite loss that occurs regardless of the antitumor effect might be one of the reasons. In our study, all patients with an increased ALBI score showed a decrease in the serum albumin level. Although the cause of this decrease in the serum albumin level is unclear, it is possible that the age of the patients influenced the results, as the median age in this study was older than that in the IMbrave150 trial (74 vs. 64 years old, respectively). It is well known that appetite loss and organ failure can easily occur in elderly patients, meaning that albumin might be easily reduced in elderly patients, even though the decrease was not so severe as to constitute an AE. Proteinuria is a major AE of bevacizumab and may be the reason for the albumin reduction; however, there was no apparent difference in the rates of proteinuria between the two studies (Table S3). As liver function sometimes worsened at an early stage, the introduction of the next treatment regimen must be decided as soon as possible.

The present study has two limitations. First, this study was retrospective and did not have a large sample size. Second, it has only been 18 months since atezolizumab plus bevacizumab was approved in Japan for the treatment of HCC, so the observation period was very short; the collection and analysis of more cases is therefore still needed.

Nevertheless, we showed in the present study that the liver function and systemic therapy are useful predictors of e‐PD in patients receiving atezolizumab plus bevacizumab in real‐world clinical practice. We hope that the information from this study will be beneficial in real‐world clinical practice.

## AUTHOR CONTRIBUTIONS

**Yasuto Takeuchi:** Conceptualization (lead); data curation (lead); formal analysis (lead); investigation (lead); methodology (lead); project administration (lead); writing – original draft (lead). **Kazuhiro Nouso:** Writing – review and editing (supporting). **Shinichi Fujioka:** Data curation (equal). **Kazuya Kariyama:** Data curation (equal). **Haruhiko Kobashi:** Data curation (equal). **Shuji Uematsu:** Data curation (equal). **Akio Moriya:** Data curation (equal). **Hiroaki Hagihara:** Data curation (equal). **Hiroyuki Takabatake:** Data curation (equal). **Shinichiro Nakamura:** Data curation (equal). **Kazuhisa Yabushita:** Data curation (equal). **Tatsuya Kikuchi:** Data curation (equal). **Atsushi Oyama:** Data curation (equal). **Takuya Adachi:** Data curation (equal). **Nozomu Wada:** Data curation (equal). **Hideki Onishi:** Data curation (equal). **Hidenori Shiraha:** Data curation (equal). **Akinobu Takaki:** Data curation (equal).

## FUNDING INFORMATION

This research does not receive any specific grant from funding agencies in the public, commercial, or not‐for‐profit sectors.

## CONFLICT OF INTEREST STATEMENT

The authors have no conflict of interest to declare.

## ETHICS STATEMENT

Written informed consent was obtained from all patients, and the study was conducted in accordance with the Declaration of Helsinki. All protocols were approved by the ethics committees of the institutes (ken‐2012‐017).

## Supporting information


Table S1.
Table S2.
Table S3.

## Data Availability

The datasets generated and/or analyzed during the current study are available from the corresponding author on reasonable request.
