# Supplementary material for: The prediction of early progressive disease in patients with hepatocellular carcinoma receiving atezolizumab plus bevacizumab
Source: Cancer Med. 2023 Aug 3;12(17):17559–68. doi: 10.1002/cam4.6369 (PMC10523973; doi:10.1002/cam4.6369)
Supplement: Supplementary file 1 — Table S1. Table S2. Table S3. [file CAM4-12-17559-s001.docx]

Supplement table 1: Sequential therapy after atezolizumab plus bevacizumab therapy

| Treatment | e-PD  (n = 50) | Non-e-PD  (n = 132) | P |
| --- | --- | --- | --- |
|  | 24 (51.1%) | 57 (43.2%) | 0.10 |
| Agent |  |  |  |
| Lenvatinib | 17 | 41 |  |
| Sorafenib | 3 | 6 |  |
| Ramucirumab | 1 | 2 |  |
| Cabozantinib | 3 | 8 |  |

Supplement table 2: Results of a logistic regression analysis of the factors associated with e-PD in patients without a systemic therapy

|  | Univariate analysis | | Multivariate analysis | |
| --- | --- | --- | --- | --- |
| Factors | Odds ratio (95% CI) | *P value* | Odds ratio (95% CI) | *P value* |
| Age (≥75 years) | 1.2 (0.50-3.1) | 0.64 |  |  |
| Etiology (NAFLD) | 2.8 (1.0-7.8) | 0.047 | 2.6 (0.88-7.8) | 0.085 |
| Performance status (≥1) | 3.7 (1.3-10) | 0.012 | 2.4 (0.80-7.2) | 0.12 |
| ALBI score (≥-2.30) | 1.5 (0.59-3.7) | 0.41 |  |  |
| Macrovascular invasion (present) | 1.8 (0.56-5.8) | 0.32 |  |  |
| Extrahepatic metastasis (present) | 2.2 (0.85-5.9) | 0.10 |  |  |
| BCLC (C) | 2.7 (0.98-7.5) | 0.049 | 2.3 (0.76-6.9) | 0.14 |
| AFP (≥400 ng/ml) | 1.1 (0.37-3.3) | 0.87 |  |  |
| AFP-L3 (≥25%) | 1.5 (0.55-4.1) | 0.42 |  |  |
| DCP (≥400 mAU/mL) | 1.4 (0.58-3.6) | 0.43 |  |  |
| AE within 9 weeks (present) | 1.0 (0.33-3.1) | 0.99 |  |  |
| Bevacizumab reduction or withdrawal within 9 weeks (present) | 1.0 (0.20-5.2) | 0.98 |  |  |

CI, confidence interval; NAFLD, nonalcoholic fatty liver disease; ALBI, albumin–bilirubin; AFP, alpha-fetoprotein; AFP-L3, LCA-reactive alpha-fetoprotein isoform; DCP, des-gamma-carboxy prothrombin; AE, adverse event

Supplement table 3: Adverse events within 9 weeks

|  | e-PD  (n = 50) | Non-e-PD  (n = 132) | P |
| --- | --- | --- | --- |
|  | 11 (22.0%) | 27 (20.5%) | 0.42 |
| Events |  |  |  |
| Proteinuria | 4 | 10 |  |
| Hypertension | 2 | 5 |  |
| Fatigue | 2 | 4 |  |
| Liver dysfunction | 1 | 2 |  |
| Diarrhea | 0 | 1 |  |
| Pyrexia | 2 | 3 |  |
| Rash | 1 | 3 |  |
| Anorexia | 1 | 2 |  |
| varicose vein rupture | 0 | 0 |  |
| Hypothyroidism | 1 | 4 |  |
